# Comparative preventive efficacy of ProHeart^®^ 12, Heartgard^®^ Plus and Interceptor^®^ Plus against a macrocyclic lactone-resistant strain (JYD-34) of heartworm (*Dirofilaria immitis*) in dogs

**DOI:** 10.1186/s13071-021-04708-3

**Published:** 2021-04-26

**Authors:** Tom L. McTier, Susan Holzmer, Kristina Kryda, Sean Mahabir, John W. McCall, Jami Trombley, Steven J. Maeder

**Affiliations:** 1 Veterinary Medicine Research and Development, 333 Portage St, Kalamazoo, MI 49007 USA; 2 TRS Labs Inc, PO Box 5112, Athens, GA 30606 USA; 3 Northern Biomedical Research Inc, 1210 Pontaluna Road, Spring Lake, MI USA

**Keywords:** *Dirofilaria immitis*, Heartworm, ProHeart^®^ 12, Heartgard^®^ Plus, Interceptor^®^ Plus, Laboratory study, Macrocyclic lactone, Moxidectin, Resistance

## Abstract

**Background:**

The current studies compared ProHeart^®^ 12, Heartgard^®^ Plus and Interceptor^®^ Plus for preventive efficacy against JYD-34, a macrocyclic lactone (ML)-resistant strain of *Dirofilaria immitis* in dogs.

**Methods:**

In two studies, each using 24 adult beagles, dogs were allocated to four treatment groups (*n* = 6): placebo-treated control; ProHeart 12 as per label (0.5 mg/kg moxidectin); Heartgard Plus (HGP) as per label (minimum 6 µg/kg ivermectin); and Interceptor Plus (INP) as per label (minimum 0.5 mg/kg milbemycin oxime). In both studies, ProHeart 12 was administered as a single subcutaneous dose on day 0, and HGP and INP were administered orally on days 0, 30, 60, 90, 120 and 150. In Studies 1 and 2, dogs were inoculated with 50 third-stage heartworm larvae (JYD-34 strain) on days −30 and 165, respectively. In Study 2, treatment for both HGP and INP was continued on days 180, 210, 240, 270, 300 and 330. Adult heartworm recoveries were performed on day 185 in Study 1 and on day 360 in Study 2.

**Results:**

In Studies 1 and 2, all placebo-treated dogs developed adult heartworm infections (geometric mean, 29.9 and 34.9 worms/dog, respectively). A single dose of ProHeart 12 was 100% effective in preventing the development of adult JYD-34 heartworms when treatment was initiated 30 days after heartworm inoculation, while six consecutive monthly doses of HGP and INP were only 10.5% and 14.6% effective, respectively. The mean worm count for the ProHeart 12-treated group was significantly lower (*P* < 0.0001) than that for the placebo control, HGP- and INP-treated groups. In Study 2, the dogs treated with ProHeart 12 had an efficacy of 98.3%. All dogs treated with HGP and INP for 12 consecutive months had adult heartworms with efficacies of 37.7% and 34.9%, respectively. The mean worm count for the ProHeart 12-treated dogs was significantly lower (*P* < 0.0001) than those for the control group, HGP- and INP-treated groups.

**Conclusions:**

A single administration of ProHeart 12 was 98–100% effective in preventing the development of the ML-resistant JYD-34 heartworm strain and was significantly better than multiple consecutive monthly doses of either Heartgard Plus or Interceptor Plus in both studies.

**Graphic Abstract:**

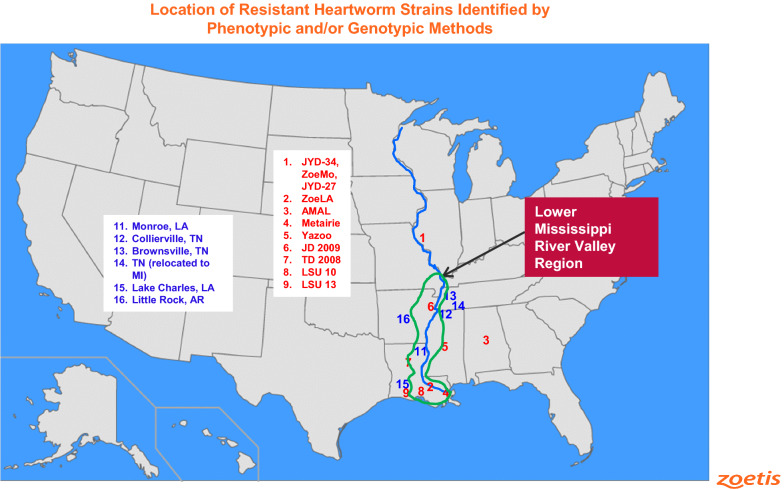

## Background

*Dirofilaria immitis*, the filarial nematode that causes heartworm disease, is one of the most important pathogenic parasites of canines globally. This parasite appears to be increasing in prevalence and expanding its range around the world due to a number of factors (e.g. continued issues with lack of or inconsistent preventive treatment, vector/disease expansion, increased animal movement, and misdiagnosis) [1–4]. A recent survey by the American Heartworm Society indicates a continued concern with heartworm disease in the United States, with 83% of clinics noting a steady or increasing incidence in 2019 [5].

The macrocyclic lactone (ML) class is the only drug class approved by regulatory authorities in heartworm preventive products, and these drugs are still highly effective in the prevention of canine heartworm disease when used as per label in most cases [6]. However, failure to completely prevent adult heartworm infection may occur despite preventive use, for several reasons. Lack of compliance with monthly preventives has been identified as a serious issue, with owners on average only administering ~ 50% of the preventive medications prescribed by their veterinarians [1, 7]. More recently, there is a growing concern with strains of heartworm that are resistant to MLs. ML class resistance has been confirmed, with more than 15 different *D. immitis* strains having been isolated from the field showing genetic and/or phenotypic evidence of ML resistance, some with dramatically reduced efficacy to the standard doses of some ML preventive products [8–13].

Of the five MLs available in approved heartworm preventive products (ivermectin, milbemycin oxime, selamectin, eprinomectin and moxidectin), moxidectin has shown the most promise in improving efficacy against various ML-resistant heartworm strains in laboratory studies in formulations and at doses and regimens designed to mimic regular preventive programs [8, 14–16]. Moxidectin has some distinguishing characteristics that make it unique for use as a heartworm preventive, especially in light of the emergence of ML resistance. Moxidectin is the most potent of the MLs, has the longest intrinsic half-life and the best pharmacokinetic profile, and is highly lipophilic, increasing its concentration in tissues where migrating heartworm larvae are likely located [17]. Increasing the oral monthly dosage of moxidectin along with multiple consecutive monthly doses has demonstrated improved efficacy against ML-resistant *D. immitis* [14, 18]. At the original approved oral dose 3 µg/kg, moxidectin was 100% effective against a susceptible heartworm strain when administered as a single oral dose 30 days after heartworm inoculation [19], while this same 3 µg/kg oral dose was only 19% effective and three successive monthly doses were only 44% effective in preventing the development of JYD-34 heartworms [14]. ProHeart^®^ 6, the same microsphere formulation as ProHeart 12 but administered at one-third the dose (0.17 mg/kg) of ProHeart 12, was 100% effective in preventing the development of a susceptible heartworm strain for 6 months; however, against a ML-resistant strain, ProHeart 6 was ineffective at the end of 6 months [10]. In contrast, when dogs were dosed at the same time as heartworm inoculation (mimicking infection at the time of initial dosing or at the end of the initial dosing interval followed by re-dosing) ProHeart 6 was 99.5% effective against a ML-resistant strain [15]. Additionally, in a large field study including ~ 300 client-owned dogs presented as veterinary patients, ProHeart^®^ 12 was 100% effective in preventing heartworm disease in the United States, including in dogs from areas where ML-resistant heartworm strains have previously been isolated [20]. All of these examples indicate that even though resistance to a drug might be occurring, there are ways to overcome resistance, at least in the short term, by increasing the dosage, the frequency of dosing or the total drug exposure using long-acting/extended-release formulations.

The current studies compared the preventive heartworm efficacy of a single subcutaneous (SC) injection of an extended-release formulation of ProHeart 12, containing 0.5 mg/kg moxidectin, to monthly doses of either Heartgard^®^ Plus (ivermectin/pyrantel) or Interceptor^®^ Plus (milbemycin oxime/praziquantel) administered orally at their approved label doses against the confirmed ML-resistant *D. immitis* strain, JYD-34. The two studies examined the efficacy of ProHeart 12 when experimental JYD-34 heartworm inoculation was conducted 30 days prior to or 165 days after treatment administration.

## Methods

### Design

The study designs are summarized in Tables 1 and 2. These placebo-controlled, comparative, masked, randomized laboratory studies were conducted according to the International Co-operation on Harmonization of Technical Requirements for Registration of Veterinary Medicinal Products (VICH) GL7, “Efficacy of Anthelmintics: General Requirements” [21] and VICH GL19 “Efficacy of Anthelmintics: Specific Recommendations for Canines” [22], and complied with VICH GL9 “Good Clinical Practice” guidelines [23].

**Table 1:** Study 1: ProHeart 12 compared to Heartgard Plus or Interceptor Plus against the JYD-34 heartworm strain: Design, day −30 heartworm inoculation

Group	Treatment	Dosage	Number of dogs	Day of L3 *D. immitis* inoculation ^a^	Days of treatment	Days of blood MF and adult *D. immitis* AG testing	Day of necropsy and adult *D. immitis* worm recovery
T01	Negative control	NA	6	−30	0	−36, 61, 121^c^, 151, 178	185 (215 days PI)
T02	ProHeart 12	0.5 mg/kg moxidectin^b^	6	−30	0		
T03	Heartgard Plus	Min. of 6 µg/kg ivermectin ^b^	6	−30	0, 30, 60, 90, 120, 150		
T04	Interceptor Plus	Min. of 0.5 mg/kg milbemycin oxime ^b^	6	−30	0, 30, 60, 90, 120, 150		

^a^Each dog was inoculated with 50 *D. immitis* third-stage larvae (JYD-34 strain)

^b^ProHeart 12, Heartgard Plus (ivermectin + pyrantel) and Interceptor Plus (milbemycin oxime + praziquantel) administered at their approved dosages according to their commercial label directions

^c^On day 121 only adult *D. immitis* AG testing was conducted

**Table 2:** Study 2: ProHeart 12 compared to Heartgard Plus or Interceptor Plus against the JYD-34 heartworm strain: Design, day 165 heartworm inoculation

Group	Treatment	Dosage	Number of dogs	Day of L3 *D. immitis* inoculation ^a^	Days of treatment	Days of blood MF and adult *D. immitis* AG testing	Day of necropsy and adult *D. immitis* worm recovery
T01	Negative control	NA	6	165	0	−14, 61, 331, 360	360 (195 days PI)
T02	ProHeart 12	0.5 mg/kg moxidectin^b^	6	165	0		
T03	Heartgard Plus	Min. of 6 µg/kg ivermectin ^b^	6	165	0, 30, 60, 90, 120, 150, 180, 210, 240, 270, 300, 330		
T04	Interceptor Plus	Min. of 0.5 mg/kg milbemycin oxime ^b^	6	165	0, 30, 60, 90, 120, 150, 180, 210, 240, 270, 300, 330		

^a^Each dog was inoculated with 50 *D. immitis* third-stage larvae (JYD-34 strain)

^b^ProHeart 12, Heartgard Plus (ivermectin + pyrantel) and Interceptor Plus (milbemycin oxime + praziquantel) administered at their approved dosages according to their commercial label directions

Personnel involved in making assessments of efficacy or safety, including physical examination, *D. immitis* third-stage larvae (L3) inoculation, treatment administration, clinical observations and recovery of adult heartworms, were masked to treatment assignments.

### Animals

Six purpose-bred beagles, both males and females, individually identified by ear tattoo, were included in each treatment group. At the time of the first treatment administration, dogs ranged in age from 12 to 15 months and body weight ranged from 6.3 to 12.7 kg. All dogs were in good health at the time of enrollment based on physical examination by a veterinarian. All dogs tested negative for blood microfilariae (MF) and adult *D. immitis* antigen (AG) prior to the L3 inoculation (Study 1) or the first treatment administration (Study 2). None of the dogs had any ML-containing products administered within 90 days prior to the start of the study and none of the dogs had ever received ProHeart 6 or ProHeart 12.

Dogs were housed indoors in mosquito-proof facility that complied with accepted animal welfare legislation and guidance. Standard environmental conditions were maintained and environmental enrichment and social interactions were provided. Dogs were fed an appropriate commercial canine maintenance diet and had access to water ad libitum. Dogs were acclimated at the facility for at least seven days prior to L3 inoculation (Study 1) or the initial treatment administration (Study 2) and were observed for general health at least once daily throughout the study.

### *Dirofilaria immitis* strain

The *D. immitis* strain, JYD-34, used in these studies was obtained from an isolate collected from a dog that was naturally infected with *D. immitis.* This dog originally came from Illinois in July 2010. This isolate was validated as an infective strain through inoculation of recipient dogs with L3 obtained by infecting mosquitoes with MF from the original donor dog, then following those recipient dogs until they were diagnosed with circulating MF, and developed positive adult heartworm antigen (AG) test results. The same generation of JYD-34 was used in both the studies and was the second passage (generation) of this strain since the original isolation in 2010. The same donor dog was used as the source of MF to infect the mosquitoes for collection of L3 for both studies. The JYD-34 strain has previously been confirmed to be resistant to MLs in multiple studies [8, 12, 24, 25].

### *Dirofilaria immitis* inoculations with L3

The L3 used for inoculation were harvested from infected *Aedes aegypti* mosquitoes reared and maintained at Zoetis (Kalamazoo, MI, USA) or TRS Labs, Inc (Athens, GA) using techniques previously described [26].

In Study 1, 30 days prior to the first treatment administration on day 0, each dog was inoculated with 50 L3 by SC injection in the inguinal region. In Study 2, 165 days after ProHeart 12 administration and 15 days after the sixth consecutive dose of either Heartgard Plus or Interceptor Plus (day 165), dogs were inoculated with 50 L3 by SC injection in the inguinal region.

### Randomization and treatments

For both studies, dogs were allocated to treatment according to a randomized complete block design with a one-way treatment structure. Dogs were ranked by day −3 (Study 1) or day −7 (Study 2) body weight to form blocks. Treatments were allocated at random to dogs within a block so that each treatment group occurred once within each block.

In Study 1, saline was administered to control dogs (T01) by SC injection on day 0. Dogs treated with ProHeart 12 (T02) received a single SC dose (0.5 mg/kg moxidectin) as per label on day 0. Dogs treated with Heartgard Plus (T03) or Interceptor Plus (T04) received the appropriate test material as per label on days 0, 30, 60, 90, 120 and 150.

In Study 2, saline was administered to control dogs (T01) by SC injection on day 0. Dogs treated with ProHeart 12 (T02) received a single SC dose (0.5 mg/kg moxidectin) as per label on day 0. Dogs treated with Heartgard Plus (T03) or Interceptor Plus (T04) received the appropriate test material as per label on days 0, 30, 60, 90, 120, 150, 180, 210, 240, 270, 300 and 330.

Heartgard Plus chewables and Interceptor Plus chewable tablets were obtained from a commercial supplier and were administered according to their approved commercial dosing instructions, which resulted in dogs treated with Heartgard Plus receiving from 6.5 to 11.5 µg/kg ivermectin and dogs treated with Interceptor Plus receiving from 0.51 to 1.1 mg/kg milbemycin oxime across both studies.

Body weights obtained within the seven days prior to each dose administration were used for dose calculation. All doses for dogs treated with Heartgard Plus and Interceptor Plus were administered by mouth, and each dog was observed for several minutes after dosing for evidence that the dose was swallowed, and for approximately 2 hours after dosing for evidence of emesis.

### Blood MF and AG testing

For Study 1, blood was collected from each dog on days −36, 61, 121, 151 and 178 for examination for blood MF and/or AG testing. On day 121, dogs were tested for *D. immitis* AG only. Testing of blood collected on days −36 and 61 was conducted to detect *D. immitis* infections that may have been present prior to experimental inoculation, and testing of samples collected on days 121, 151 and 178 was conducted to detect infection from the experimental *D. immitis* inoculation. For Study 2, blood was collected on days −11, 63, 331 and 360 for examination for blood MF and/or AG testing. Testing of blood collected on days −11 and 63 was conducted to detect *D. immitis* infections that may have been present prior to experimental inoculation, and testing of samples collected on days 331 and 360 was conducted to detect infection from the experimental *D. immitis* inoculation conducted on day 165.

A modified Knott’s procedure was used for the blood MF examination and a commercially available test kit (DiroCHEK Heartworm AG Test Kit, Zoetis, Parsippany, NJ) was used for detection of AG.

Heat treatment of some samples of plasma was conducted to confirm the heartworm infection status of dogs. The method used was that as described by Little et al. [27].

### Health observations

Dogs were examined for general health twice daily except when clinical observations were conducted. Clinical observations were conducted on all dogs prior to and at 1, 3, 6 and 24 hours after each treatment administration.

### Necropsy and adult *D. immitis* worm recovery

Approximately 7 months after inoculation of dogs with L3, on day 185 (215 days post-inoculation [PI]) for Study 1 and on day 360 (195 days PI) for Study 2, all dogs were humanely euthanized by administration of an intravenous heparin/pentobarbital solution administered according to the approved label directions. The pleural and peritoneal cavities were examined for adult *D. immitis*, the posterior and anterior vena cavae were clamped, and the heart and lungs were removed. The precava, right atrium, right ventricle and pulmonary arteries (including those coursing through the lungs) were dissected and examined. Any adult worms present were recovered and classified as male or female and as either dead (worms abnormal in both appearance and motility) or alive (all other worms). “Abnormal” means that, in comparison to worms from placebo-treated dogs, these worms were structurally different in appearance (color, size, body integrity) and motility (worm movement upon visualization in warm media which stimulates movement). Dogs were randomly assigned to order of euthanasia and necropsy.

### Statistical analysis

The experimental unit for treatment was the individual animal for Study 1 and the pen for Study 2. The primary endpoint for both studies was the total (live + dead) worm count. Worm counts were transformed by the ln (count +1) transformation prior to analysis in order to stabilize the variance and normalize the data. Transformed counts were analyzed using a mixed linear model (SAS 9.3, Cary NC) that included the fixed effect of treatment, and the random effects of block, interaction between block and treatment, and error. Testing was two-sided at the significance level α = 0.05.

Percent efficacy relative to placebo control was calculated using geometric means (back-transformed least square means) based on the formula [(C − T)/C] × 100, where C = mean worm count for the placebo group and T = mean worm count for the treated group.

## Results

### MF and AG testing

All dogs were negative for blood MF and AG on days −36 and 61 (Study 1), and −11 and 63 (Study 2), confirming that none of the dogs was infected with heartworms prior to inoculation for the study.

In Study 1, on days 151 (181 days PI) and 178 (208 days PI), all placebo-treated dogs and all Interceptor Plus-treated dogs were AG-positive, even though some dogs were positive only after heat treatment of samples (Table 3). All dogs treated with Heartgard Plus were AG-positive on both days 151 and 178, except for a single dog on day 151 that remained AG-negative even after heat treatment. On day 178 for placebo, Heartgard Plus and Interceptor Plus groups, all dogs were microfilaremic. All dogs treated with ProHeart 12 were both AG- and MF-negative on both days 151 and 178.

**Table 3:** Study 1: Efficacy of ProHeart 12 compared to Heartgard^®^ Plus or Interceptor^®^ Plus against the JYD-34 heartworm strain: Day −30 inoculation, adult heartworm AG and MF results

Treatment	Dosage	Days of treatment	Number of infected dogs^a, b^	No. dogs positive for adult heartworm AG^c^		No. dogs positive for MF	
				Day 151 (181 days PI)	Day 178 (208 days PI)	Day 151 (181 days PI)	Day 178 (208 days PI)
Negative control	NA	0	6 of 6	6 of 6	6 of 6	4 of 6	6 of 6
ProHeart 12	0.5 mg/kg moxidectin^d^	0	0 of 5	0 of 5	0 of 5	0 of 5	0 of 5
Heartgard Plus	Min. of 6 µg/kg ivermectin^e^	0, 30, 60, 90, 120, 150	6 of 6	5 of 6^f^	6 of 6^g^	5 of 6	6 of 6
Interceptor Plus	Min. of 0.5 mg/kg milbemycin oxime^h^	0, 30, 60, 90, 120, 150	6 of 6	6 of 6	6 of 6	6 of 6	6 of 6

^a^Each dog was inoculated with 50 *D. immitis* third-stage larvae (JYD-34 strain) on day −30

^b^All dogs were necropsied for recovery of adult *D. immitis* on day 185 (215 days PI; 35 days after the last treatment with Heartgard^®^ Plus and Interceptor^®^ Plus)

^c^DiroCHEK

^d^ProHeart 12 (moxidectin extended-release injectable suspension) administered according to commercial label directions which resulted in a point administration of 0.5 mg/kg of moxidectin to each dog. One dog in the ProHeart 12 group was euthanized on day 90 due to an abnormal health event unrelated to drug treatment

^e^Heartgard Plus (ivermectin + pyrantel) administered according to commercial label directions which resulted in administration of from 6.6 to 11.2 µg/kg of ivermectin

^f^Two of six dogs positive only after heat treatment; positive on both DiroCHEK and Snap4DX Plus

^g^Two of six dogs positive only after heat treatment; positive on both DiroCHEK and Snap4DX Plus

^h^Interceptor Plus (milbemycin oxime + praziquantel) administered according to commercial label directions which resulted in administration of from 0.52 to 1.0 mg/kg milbemycin oxime

In Study 2, on both days 331 (166 days PI) and 360 (195 days PI), all dogs treated with either placebo, Heartgard Plus or Interceptor Plus were AG-positive, although some dogs were positive only after heat treatment of samples (Table 4). In addition, all dogs in all groups were amicrofilaremic on day 331, while five of six dogs from each of the placebo, Heartgard Plus and Interceptor Plus groups were positive for MF on day 360. For ProHeart 12-treated dogs, none was positive for AG on day 331, while one dog with a single male worm was slightly positive on day 360 after sample heat treatment. All ProHeart 12-treated dogs were negative for MF on both days 331 and 360. All MF samples were indicated as positive or negative for the presence of MF, and the numbers of MF were not quantified.

**Table 4:** Study 2: Efficacy of ProHeart 12 compared to Heartgard Plus or Interceptor Plus against the JYD-34 heartworm strain: Day 165 inoculation, adult heartworm AG and MF results

Treatment	Dosage	Days of treatment	Number of infected dogs^a, b^	No. dogs positive for adult heartworm AG^c^		No. dogs positive for MF	
				Day 331 (day 166 PI)	Day 360 (day 195 PI)	Day 331 (day 166 PI)	Day 360 (day 195 PI)
Negative control	NA	0	6 of 6	6 of 6^d^	6 of 6^e^	0 of 6	5 of 6
ProHeart 12	0.5 mg/kg moxidectin^f^	0	4 of 6	0 of 6^g^	1 of 6^h^	0 of 6	0 of 6
Heartgard Plus	Min. of 6 µg/kg ivermectin^i^	0, 30, 60, 90, 120, 150, 180, 210, 240, 270, 300, 330	6 of 6	6 of 6^j^	6 of 6	0 of 6	5 of 6
Interceptor Plus	Min. of 0.5 mg/kg milbemycin oxime^k^	0, 30, 60, 90, 120, 150, 180, 210, 240, 270, 300, 330	6 of 6	6 of 6^l^	6 of 6^m^	0 of 6	5 of 6

^a^Each dog was inoculated with 50 *D. immitis* third-stage larvae (JYD-34 strain) on day 165

^b^All dogs were necropsied for recovery of adult *D. immitis* on day 360 (195 days PI; 30 days after the last treatment with Heartgard Plus and Interceptor Plus)

^c^DiroCHEK

^d^ Two of six dogs positive only after heat treatment; positive on both DiroCHEK and Snap4DX

^e^One of six dogs positive only after heat treatment; positive on both DiroCHEK and Snap4DX

^f^ ProHeart 12 (moxidectin extended-release injectable suspension) administered according to commercial label directions which resulted in a point administration of 0.5 mg/kg of moxidectin to each dog

^g^All six of the dogs remained Ag-negative after samples were heat treated

^h^One dog with a single male worm was slightly positive only after heat treatment; positive on both DiroCHEK and Snap4DX

^i^Heartgard Plus (ivermectin + pyrantel) administered according to commercial label directions which resulted in administration of 6.5 to 11.5 µg/kg of ivermectin

^j^Three of six dogs positive only after heat treatment; positive on both DiroCHEK and Snap4DX

^k^Interceptor Plus (milbemycin oxime + praziquantel) administered according to commercial label directions which resulted in administration of 0.51 to 1.1 mg/kg milbemycin oxime

^l^Five of six dogs positive only after heat treatment; positive on both DiroCHEK and Snap4DX

^m^One of six dogs positive only after heat treatment; positive on both DiroCHEK and Snap4DX

### Health observations

There were no treatment-related adverse reactions in the study. In general, observations of abnormal health were minor, and their incidence was generally similar between treatment groups. Most abnormal health events were of the type commonly observed in laboratory dogs, and included gastrointestinal, dermatologic and ophthalmic abnormalities.

One dog in Study 1 in the ProHeart 12-treated group had a grand mal seizure on day 90, was treated with intravenous midazolam and became stable. The following morning upon examination, the dog appeared to be postictal, and cluster seizures during the night were suspected. Due to welfare concerns, the dog was humanely euthanized. A full diagnostic necropsy with a board-certified pathologist was performed but was unremarkable, and a diagnosis of idiopathic epilepsy was made, likely unrelated to treatment, as treatment was administered 90 days previously, and peak plasma levels for ProHeart 12 are achieved approximately 2 weeks post-treatment (PT).

### Efficacy in preventing the development of the ML-resistant JYD-34 heartworm strain

In Study 1, all six placebo-treated dogs were infected with adult heartworms at necropsy, with a geometric mean of 29.9 heartworms per dog and a range of 23–37 worms (Table 5). All ProHeart 12-treated dogs were free of adult heartworms, indicating that ProHeart 12 was 100% effective in preventing the development of the JYD-34 strain in dogs when treatment was administered 30 days PI with L3. All six dogs treated with six consecutive doses of Heartgard Plus were infected with adult heartworms, with a geometric mean of 26.8 worms (range, 19–34), and all six dogs treated with six consecutive doses of Interceptor Plus were also infected, with a geometric mean of 25.5 worms (range, 13 to 35). This resulted in percentage efficacy of only 10.5% and 14.6% for Heartgard Plus and Interceptor Plus, respectively. The geometric mean worm count for the ProHeart 12-treated dogs was significantly lower (*P* < 0.0001) than that for the control group and for both Heartgard Plus and Interceptor Plus. Geometric mean counts for the Heartgard Plus- and Interceptor Plus-treated groups were not significantly different from the control group (*P* ≥ 0.2719) or each other (*P* = 0.7405) (Table 5).

**Table 5:** Study 1: Efficacy of ProHeart 12 compared to Heartgard Plus or Interceptor Plus against the JYD-34 heartworm strain: Day −30 inoculation, adult heartworm counts and percentage reductions

Treatment	Dosage	Days of treatment	Number of infected dogs ^a^	Adult *D. immitis* worm counts		Efficacy compared to negative control ^c^
				Individual worm counts	Geometric mean ^b^	Percentage reduction
Negative control	NA	0	6 of 6	23, 25, 29, 33, 35, 37	29.9^g^	NA
ProHeart 12	0.5 mg/kg moxidectin ^d^	0	0 of 5	0	0^h^	100
Heartgard Plus	Min. of 6 µg/kg ivermectin ^e^	0, 30, 60, 90, 120, 150	6 of 6	19, 21, 24, 33, 34, 34	26.8^g^	10.5
Interceptor Plus	Min. of 0.5 mg/kg milbemycin oxime ^f^	0, 30, 60, 90, 120, 150	6 of 6	13, 24, 27, 30, 31, 35	25.5^g^	14.6

^a^Each dog was inoculated with 50 *D. immitis* third-stage larvae (JYD-34 strain) on day −30

^b^Geometric mean counts with different superscript letters are significantly different (*P* < 0.0001), see ^g, h,^

^c^All dogs were necropsied for recovery of adult *D. immitis* on day 185 (215 days PI; 35 days after the last treatment with Heartgard Plus and Interceptor Plus)

^d^ProHeart 12 (moxidectin extended-release injectable suspension) administered according to commercial label directions which resulted in a point administration of 0.5 mg/kg of moxidectin to each dog. One dog in the ProHeart 12 group was euthanized on day 90 due to an abnormal health event unrelated to drug treatment

^e^Heartgard Plus (ivermectin + pyrantel) administered according to commercial label directions which resulted in administration of from 6.6 to 11.2 µg/kg of ivermectin

^f^Interceptor Plus (milbemycin oxime + praziquantel) administered according to commercial label directions which resulted in administration of from 0.52 to 1.0 mg/kg milbemycin oxime

Similarly, in Study 2, all six negative control placebo-treated dogs were infected with adult heartworms, with a geometric mean of 34.9 heartworms per dog and a range of 27 to 46 worms (Table 6). Two of the six ProHeart 12-treated dogs were completely free of adult heartworms, with the remaining four dogs each having a live single worm. This resulted in an overall efficacy of 98.3% in preventing the development of the JYD-34 strain when dogs were inoculated with L3 larvae 165 days after treatment with ProHeart 12. All six dogs treated with 12 consecutive doses (6 doses prior to L3 inoculation and 6 doses after L3 inoculation) of Heartgard Plus were infected with adult heartworms with a geometric mean of 21.7 worms (range, 12 to 29), and all six dogs treated with 12 consecutive doses of Interceptor Plus were also infected with a geometric mean of 22.7 worms (range, 14 to 37). This resulted in similar percentage efficacy of only 37.7% and 34.9% for Heartgard Plus and Interceptor Plus, respectively. The geometric mean worm count for the ProHeart 12-treated dogs was significantly lower (*P* < 0.0001) than those for the control group and for both Heartgard Plus and Interceptor Plus. Geometric mean counts for Heartgard Plus or Interceptor Plus were also significantly different from the control group (*P* ≥ 0.0321) but not each other (*P* = 0.8147). All heartworms collected from all dogs were alive and of similar size.

**Table 6:** Study 2: Efficacy of ProHeart 12 compared to Heartgard Plus or Interceptor Plus against the JYD-34 heartworm strain: Day 165 inoculation, adult heartworm counts and percentage reductions

Treatment	Dosage	Days of treatment	Number of infected dogs^a^	Adult *D. immitis* worm counts		Efficacy compared to negative control ^c^
				Individual worm counts	Geometric mean^b^	Percentage reduction
Negative control	NA	0	6 of 6	27, 32, 34, 36, 37, 46	34.9^g^	NA
ProHeart 12	0.5 mg/kg moxidectin ^d^	0	4 of 6	0, 0, 1, 1, 1, 1	0.6^h^	98.3
Heartgard Plus	Min. of 6 µg/kg ivermectin^e^	0, 30, 60, 90, 120, 150, 180, 210, 240, 270, 300, 330	6 of 6	12, 20, 23, 24, 27, 29	21.7^i^	37.7
Interceptor Plus	Min. of 0.5 mg/kg milbemycin oxime^f^	0, 30, 60, 90, 120, 150, 180, 210, 240, 270, 300, 330	6 of 6	14, 19, 23, 24, 25, 37	22.7^i^	34.9

^a^Each dog was inoculated with 50 *D. immitis* third-stage larvae (JYD-34 strain) on day 165

^b^Geometric mean counts with different superscript letters are significantly different (*P* ≤ 0.0321), see ^g, h, i^

^c^All dogs were necropsied for recovery of adult *D. immitis* on day 360 (195 days PI; 30 days after the last treatment with Heartgard Plus and Interceptor Plus)

^d^ProHeart 12 (moxidectin extended-release injectable suspension) administered according to commercial label directions which resulted in a point administration of 0.5 mg/kg of moxidectin to each dog

^e^Heartgard Plus (ivermectin + pyrantel) administered according to commercial label directions which resulted in administration of 6.5 to 11.5 µg/kg of ivermectin

^f^Interceptor Plus (milbemycin oxime + praziquantel) administered according to commercial label directions which resulted in administration of 0.51 to 1.1 mg/kg milbemycin oxime

## Discussion

In this direct comparative study, Heartgard Plus and Interceptor Plus were chosen as the comparator products for ProHeart 12, as they contain two different MLs, ivermectin and milbemycin oxime, respectively, which were the first two MLs originally approved for heartworm prevention. Additionally, the most data are available on these two MLs using various ML-resistant strains of *D. immitis*, especially the JYD-34 strain.

ProHeart 12 was 100% effective in preventing the development of the ML-resistant JYD-34 heartworm strain in dogs when a single SC treatment was administered 30 days after inoculation of L3 (Study 1) and also was highly (98.3%) effective when dogs were inoculated near the midpoint of the dosing interval (day 165, Study 2). In contrast, both Heartgard Plus and Interceptor Plus were ineffective in preventing the establishment of adult JYD-34 infections in dogs, even after six consecutive monthly doses when treatment was initiated 30 days PI (Study 1) or when treatment was initiated 6 months prior to heartworm inoculation and continued for 6 months after inoculation (Study 2). The efficacy seen in Study 1 (10.5% for Heartgard Plus (ivermectin + pyrantel) and 14.6% for Interceptor Plus (milbemycin oxime + praziquantel) was lower than that observed in previous studies with these products against JYD-34 [8, 16]. Differences in the efficacy response between studies can be explained by normal variability across studies and the genetic bottlenecking that occurs with subsequent passaging of generations of parasite strains [28, 29]. However, all dogs were infected with multiple male and female worms in all studies, confirming the phenotypic resistance of JYD-34 to these approved doses of ivermectin (minimum of 6 µ/kg) and milbemycin oxime (minimum of 0.5 mg/kg). The efficacy observed in Study 2 was somewhat higher than that observed in Study 1 at 37.7% and 34.9%, for Heartgard Plus and Interceptor Plus, respectively. However, the dosing regimen was different in this study with six monthly doses administered prior to heartworm inoculation and six monthly doses after inoculation. Additionally, the last treatment administered before heartworm inoculation was 15 days before L3 injection and the first treatment following L3 injection was 15 days after this L3 inoculation. This could account for the improved efficacy compared to Study 1, in which no doses were administered prior to inoculation and the first dose after inoculation was 30 days PI. It should be noted that the same generation (second passage) of JYD-34 from the same donor dog was used in both the studies reported in this paper. For comparison, the generation of JYD-34 used in the paper by Blagburn et al. [8] was from the original first generation of this heartworm strain established in laboratory dogs. The efficacies reported for three successive monthly doses of Heartgard Plus (ivermectin/pyrantel) and Trifexis^®^ (milbemycin oxime/spinosad) were, 29.0% and 52.2%, respectively, when treatments were initiated 30 days after inoculation of third-stage larvae.

Not only were Heartgard Plus and Interceptor Plus not effective in preventing the development of adult heartworms, they also did not prevent dogs from developing MF or, once MF-positive, in suppressing MF with ongoing treatment once infections became patent. This is especially noteworthy, as dogs infected with ML-resistant heartworms and treated in this manner could be a source of ML-resistant MF for transmission to other dogs and may act as potential reservoirs for perpetuation and spread of ML-resistant strains in the population at large. In a separate study reported in 2017, both ProHeart 6 and ProHeart 12 demonstrated high microfilaricidal activity in dogs infected with a ML-resistant strain (ZoeMO), with > 90% reduction in MF achieved at 42 days PT, with both products achieving > 96% reduction in MF at 84 days PT [30]. This underscores the need for dogs to be placed on heartworm preventives that are effective against the widest range of heartworm strains and stages (MF and larvae) to which most dogs are likely to be exposed in natural pet owner situations.

Additionally, some of the dogs treated with Heartgard Plus or Interceptor had positive AG results only after heat treatment of the plasma samples. This occurred in dogs with multiple female worms. This is not surprising as treatment with MLs does have an effect on suppressing antigen production, the majority of which is produced by the female heartworms [31–33].

The studies reported here are the first studies demonstrating the efficacy of ProHeart 12 in preventing the development of any ML-resistant heartworm strains in dogs. ProHeart 6, an extended-release injectable suspension using the exact same formulation as ProHeart 12 but at one-third the dose (0.17 mg/kg) of ProHeart 12, was 99.5% effective against the same JYD-34 strain, when treatment was administered 2 days before inoculation with L3 [15]. Additionally, ProHeart 12 was 100% effective in a large multi-site field study involving ~ 300 dogs from 19 clinics [14] across the United States, with 142 cases from the southern US region and 42 of the cases from the Lower Mississippi River Valley (LMRV) where strains of heartworm resistant to MLs have been identified [20].

These data using the ProHeart 12 injectable microsphere formulation support prior data indicating that moxidectin, based on its unique physicochemical properties (i.e. potency, longer half-life, higher lipophilicity) appears to provide enhanced activity, compared to other MLs, against ML-resistant heartworms in both topical (Advantage Multi) [8] and oral formulations (Simparica Trio) [14, 16] in comparative laboratory studies. In addition, Simparica Trio, providing 24 µg/kg moxidectin in an oral formulation, was 100% effective in preventing heartworm disease in canine veterinary patients (*n* = 246) from 23 clinics across the United States, with 16 of these clinics from the southern US region and five from the LMRV [18].

ProHeart 12 is designed to provide heartworm prevention for a full 12 months. The current studies were designed to assess efficacy against a ML-resistant strain, if dogs were to be infected prior to the beginning of the treatment (30 days) or at or near the end of the treatment interval and then re-treated (Study 1) or near the mid-point of the dosing interval (Study 2).

## Conclusions

A single administration of ProHeart 12 (0.5 mg/kg moxidectin) extended-release injectable was 100% effective in preventing adult heartworm when dogs were inoculated with heartworm larvae 30 days prior to treatment and was > 98% effective when dogs were inoculated with heartworm larvae 165 days after treatment with the ML-resistant JYD-34 strain. In these studies, ProHeart 12 was significantly better than either Heartgard Plus or Interceptor Plus, when inoculated either 30 days prior to six successive monthly doses or after six successive monthly doses, and subsequent treatment with six additional doses.

## Data Availability

The datasets supporting the conclusions of this article are included within the article.

## Abbreviations

AG: Adult heartworm antigen
LMRV: Lower Mississippi River Valley
L3: Third-stage larvae
MF: Microfilariae
ML: Macrocyclic lactone
PI: Post-inoculation
PT: Post-treatment
SC: Subcutaneous
